# “The Roaring Adventures of Puff” (RAP) – a school based asthma education program for children with asthma

**DOI:** 10.1186/1710-1492-7-S2-A20

**Published:** 2011-11-14

**Authors:** Lesley Stewart, Cathy Gillespie, Shauna Filuk, Bev Kulbaba, Jo-Anne St Vincent, Nancy Ross, Isabel Gardziel, Anna Drewniak, Allan B Becker

**Affiliations:** 1 Children’s Asthma Education Centre, Children’s Hospital, Winnipeg, Manitoba, Canada, R3E 0Z2

## Background

Asthma guidelines recognize asthma education as “an essential component of asthma therapy”. The Children’s Asthma Education Centre (CAEC)’s small group interactive sessions for children and families does not meet the needs of all families. Schools provide an opportunity for asthma education. The “Roaring Adventures of Puff” (RAP) is an effective, previously validated asthma education program.

## Methods

We conducted RAP as 6 weekly, one-hour sessions for 7-11 year old children in schools in Manitoba. Questionnaire data were collected for each child before and 6 months after RAP. The primary outcome parameter was school absenteeism, (year before vs. year after). Secondary outcomes included child and caregiver quality of life and caregiver work productivity.

## Results

The study was conducted in 25 schools in Winnipeg and 2 rural schools (n=194 students pre- and n=177 students post-intervention). Data for the Winnipeg schools are complete. The reduction in the number of missed school days in the year following RAP was significant (p<0.05). Quality of life for both the child and the parent was significantly improved (p<0.001), as was productivity for the primary caregiver at home (p<0.05). (Table 1).

**Table 1:** 

	PRE	POST
**Number of children**	194	177
**Age (years)**	8.00 (5-12)	9.00 (6-13)^1^
**Days absent from school**	8.43 ± 11.90	6.48±6.41*

^1^ - mean (range) * - mean±**SD, P <0.05**

## Conclusions

A school-based asthma education program has significant and clinically relevant benefits for the child, family and school. We strongly encourage adoption of RAP as a school based education program for children with asthma.

